# The Bilingual Disadvantage in Speech Understanding in Noise Is Likely a Frequency Effect Related to Reduced Language Exposure

**DOI:** 10.3389/fpsyg.2016.00678

**Published:** 2016-05-13

**Authors:** Jens Schmidtke

**Affiliations:** Michigan State University, East LansingMI, USA

**Keywords:** speech understanding in noise, bilingual, working memory, frequency effect, spoken word recognition

## Abstract

The present study sought to explain why bilingual speakers are disadvantaged relative to monolingual speakers when it comes to speech understanding in noise. Exemplar models of the mental lexicon hold that each encounter with a word leaves a memory trace in long-term memory. Words that we encounter frequently will be associated with richer phonetic representations in memory and therefore recognized faster and more accurately than less frequently encountered words. Because bilinguals are exposed to each of their languages less often than monolinguals by virtue of speaking two languages, they encounter all words less frequently and may therefore have poorer phonetic representations of all words compared to monolinguals. In the present study, vocabulary size was taken as an estimate for language exposure and the prediction was made that both vocabulary size and word frequency would be associated with recognition accuracy for words presented in noise. Forty-eight early Spanish–English bilingual and 53 monolingual English young adults were tested on speech understanding in noise (SUN) ability, English oral verbal ability, verbal working memory (WM), and auditory attention. Results showed that, as a group, monolinguals recognized significantly more words than bilinguals. However, this effect was attenuated by language proficiency; higher proficiency was associated with higher accuracy on the SUN test in both groups. This suggests that greater language exposure is associated with better SUN. Word frequency modulated recognition accuracy and the difference between groups was largest for low frequency words, suggesting that the bilinguals’ insufficient exposure to these words hampered recognition. The effect of WM was not significant, likely because of its large shared variance with language proficiency. The effect of auditory attention was small but significant. These results are discussed within the Ease of Language Understanding model (Rönnberg et al., 2013), which provides a framework for explaining individual differences in SUN.

## Introduction

Spoken language comprehension is a complex process that entails encoding an acoustic signal, matching it to the right phonological representation stored in long-term memory (LTM) out of thousands of such representations, and finally retrieving the semantic information associated with the phonological information and integrate it with the preceding information. Yet understanding spoken language under optimal listening conditions is usually a seemingly effortless process. Only when it comes to listening to speech under suboptimal conditions do we become conscious of this process and individual differences in people’s ability to understand speech become obvious. This is especially true in a second language, as many second language speakers can attest to and has also been shown in many studies (for a review see Garcia Lecumberri et al., 2010). What is surprising is that even speakers who learned their second language early in life and became dominant in that language still show poorer performance on speech understanding in noise (SUN) tests (Mayo et al., 1997; von Hapsburg et al., 2004; Rogers et al., 2006; Shi, 2010). To explain these findings the present study tested the hypothesis that bilinguals are disadvantaged in SUN because of their reduced exposure to each of their languages relative to monolinguals. Its contribution to the current discussion on bilingual SUN is a larger sample size of early bilingual speakers compared to previous studies and the presentation of a framework to explain bilingual disadvantages in auditory language comprehension.

The ease of language understanding (ELU) model (Rönnberg et al., 2013) provides a framework for explaining the effects of a suboptimal speech signal on listening effort. The model assumes that during listening sublexical information at the level of the syllable is buffered in a temporary storage system called RAMBPHO (rapid, automatic, multi-modally bound phonological representations). These syllabic units are then compared to phonological representations in LTM. The model assumes that phonological representations consist of multiple attributes and for successful lexical access the speech signal has to activate a minimum number of attributes. If the threshold for lexical retrieval is not reached, similar sounding words may be retrieved instead. However, contextual information may often be sufficient for a lexical item to be retrieved even when the bottom-up information from the speech signal is insufficient. In such cases where information in RAMBPHO cannot be matched to a LTM representation, explicit processing that involves working memory (WM) is needed to resolve the mismatch, causing a delay in lexical access. Otherwise lexical access occurs automatically. Mismatches between the speech signal and LTM representations can occur for speaker-external (e.g., distorted speech or an unfamiliar accent) or speaker-internal reasons (imprecise phonological representations; Rönnberg et al., 2013, p. 3).

The degree of similarity between the acoustic signal and an internal phonological representation determines the amount of processing that is needed for lexical access to be successful. When the match is optimal, processing is automatic and effortless. The greater the mismatch, the greater is the need for explicit processing of the signal. This explicit processing loop is dependent on WM resources. Thus, according to the model, individual differences in SUN can be attributable to two sources, individual differences in WM capacity and individual differences in the quality of speaker-internal phonological representations of words in LTM.

How can we explain differences in the quality of phonological representations? Exemplar models of the mental lexicon (Klatt, 1979; Goldinger, 1996, 1998; Pierrehumbert, 2000, 2003; Hawkins, 2003; Johnson, 2005) may be especially useful here. In contrast to models that assume that words in the mental lexicon are stored in an abstract form without any indexical information (e.g., speaker voice characteristics such as gender, age, etc.), exemplar models assume that each encounter with a word token leaves a separate episodic trace in memory. Thus pronunciation variants and reduced forms, for example, are also assumed to be stored (e.g., Pierrehumbert, 2001; Pitt et al., 2011). Phonetic categories are not understood as discrete symbols but as distributions in a multidimensional space that develop through experience. With increased experience, listeners develop selective attention (c.f. Nosofsky, 1986) to those acoustic-phonetic dimensions that are relevant in a given language. In these models, the effect of word frequency arises from the assumption that words that are encountered often are represented with more exemplars on a “cognitive map” than infrequent words (Pierrehumbert, 2003). During retrieval, all exemplars with a certain degree of similarity to an acoustic signal receive activation. Thus frequently reoccurring units of speech (e.g., words) receive more activation since they are associated with more exemplars. This gives high frequency words an advantage over low frequency words in terms of speed of lexical access. Furthermore, the selection of high frequency words will be more robust when information in the acoustic signal is missing or when there is noise in the signal. However, it is not just the mere frequency with which words are encountered that determines the robustness of a representation. For example, research shows that variability in the signal as it occurs through different speakers helps infants extract the distribution of phonetic categories from the signal so that minimal pairs (e.g., *buk* and *puk*) sound less similar, presumably because variability directs infants’ attention to the relevant dimension that distinguishes the minimal pairs (in this case voice onset time; Rost and McMurray, 2009). Exemplar models can also be extended to explain second language (L2) speech perception (Hardison, 2003, 2012). Because the acoustic-phonetic space is arbitrarily divided into phonetic categories that differ from language to language, listeners need to create new categories when learning a L2. Proponents of an exemplar-based mental lexicon assume that phonetic differences between a first language (L1) and a L2 can be perceived; however, at first old category labels will continue to be activated by L2 input. Again, acoustic variability in the signal may help the L2 learner create new phonetic categories by directing his attention to those dimensions that may be irrelevant in the L1 but vary systematically in the L2. For example, Japanese listeners need to learn to attend to the third formant (the third resonance peak of the vocal tract) to differentiate between American English /r/ and /l/ because this dimension is not relevant in their first language (Lotto et al., 2004). Perceptual training studies of the /r/-/l/ distinction with native Japanese speakers showed superior identification ability between the two phonetic categories when training stimuli were spoken by multiple speakers compared to a condition with a single speaker (Lively et al., 1993; Hardison, 2003, 2005). Also relevant for the present discussion is a finding from a study on second language vocabulary learning. Native English speakers who learned new Spanish words spoken by six different speakers showed better retention and faster retrieval of those words compared to those who heard the novel words spoken by one speaker only (Barcroft and Sommers, 2005; also see Sommers and Barcroft, 2011). These findings suggest that token frequency of words in the input determine the quality of mental representations of words. Multiple exemplars associated with one word will make the retrieval of that word more efficient and robust.

Within the account described above, the assumption is made that the quality of phonological representations differs within and between speakers. Within speakers they differ because high frequency words are represented with more phonetic detail than low frequency words, and between speakers because some speakers have more language experience (i.e., more exposure) than others. These assumptions are similar to the lexical quality hypothesis developed by Perfetti and Hart (2002) and Perfetti (2007) to explain individual differences in reading comprehension. A further assumption made here is that bilingual speakers differ in language experience in one language from monolinguals because they speak and hear each of their languages less often compared to someone who only speaks one language. This assumption is expressed in the weaker-links hypothesis developed by (Gollan et al., 2002, 2005, 2008) to explain differences in lexical access between monolinguals and bilinguals (see Ivanova and Costa, 2008; Diependaele et al., 2013; Cop et al., 2015). As a result of reduced language experience, all words in a bilingual’s mental lexicon will be of lower experienced frequency compared to a monolingual speaker. Frequency effects in general are pervasive in language processing (Ellis, 2002). Word frequency in particular affects lexical retrieval times (e.g., Oldfield and Wingfield, 1965; Murray and Forster, 2004) and recognition accuracy for words presented in noise (Howes, 1957). Frequency effects are logarithmic in nature, which means that changes in frequency at the low end affect lexical retrieval times and recognition accuracy more than changes at the high end (Murray and Forster, 2004). As a consequence, reduced language exposure will especially affect low frequency words. In one study, Kuperman and Van Dyke (2013) asked subjects with more and less education to rate words for their subjective, or experienced, frequency. When comparing the two groups, subjective ratings for words that are highly frequent in the language (based on a corpus count) were very similar but the lower the objective frequency, the more subjective frequency ratings of both groups diverged. This suggests that frequency estimates that are based on large corpora such as SUBTLEX (Brysbaert and New, 2009) may overestimate the frequency with which certain words are encountered for individuals with less language experience such as bilinguals. Thus the idea behind the weaker-links hypothesis and similar theories is that slower verbal processing in bilinguals is a frequency effect. Bilinguals encounter all words less frequently compared to monolinguals and so they process all words more slowly. Less efficient spoken word recognition has been shown for late and also early bilinguals (e.g., Weber and Cutler, 2004; Schmidtke, 2014).

While occurrence counts in large corpora of language can give us an idea of the relative quality of representations of words in memory (the less frequent a word the less precise its representation), it is more difficult to estimate the overall language experience of individuals. Different means of data collection are possible such as asking participants to keep a diary of daily interactions for a week or similar techniques. However, these measures are based on self-report and do not capture language experience over longer periods of time. In this paper, the assumption is made that vocabulary knowledge, and more precisely productive vocabulary knowledge, closely resembles language experience and thus the quality of phonological representations. Individuals who are able to recall infrequent words must have been exposed to these words more often than someone who is not able to recall low frequency words. Someone with a weaker phonological representation of a word may be able to recall the first sound or a similar sounding word but lexical retrieval may not be successful. This phenomenon is usually referred to as a tip-of-the-tongue state in the literature (Brown and McNeill, 1966). A second reason for not knowing a word is that the participant may have never encountered the word before. This would also suggest reduced language experience because the more someone is exposed to language, the more likely they are to encounter an infrequent word (Kuperman and Van Dyke, 2013). The prediction is then that individuals with a higher score on a vocabulary test will be overall more accurate on a word-recognition-in-noise test, and the difference compared to someone with a lower vocabulary score will be most pronounced for low frequency words. The frequency effect might thus explain why SUN in a L2 is usually more difficult compared to one’s first language and why this effect is modulated by experience in the L2 (e.g., Mayo et al., 1997; Rogers et al., 2006; Shi, 2009, 2010; Shi and Sánchez, 2010). At the same time, the frequency effect could also explain individual differences between monolingual speakers that have been shown to exist in normal hearing subjects (see Tamati et al., 2013).

As mentioned before, in the ELU there are two sources for speaker-internal individual differences in SUN. One source is the quality of internal phonological representations, as described above. The other source is differences in WM capacity. When mismatches between the acoustic signal and phonological representations occur, speech processing relies more on explicit processes, which presumably are more susceptible to individual differences in processing resources than implicit processes. Examples of such explicit processes include “inference making, semantic integration, switching of attention, storing of information, and inhibiting irrelevant information” (Rönnberg et al., 2013, p. 3). Individuals with greater WM capacity have more resources available for such processes and are thus better able to make up for missing information from the speech signal. In support of this hypothesis, studies have established a link between the quality of sensory information and maintenance of such information in WM. For instance, hearing verbal stimuli under suboptimal listening conditions leads to reduced recall accuracy of such stimuli even when intelligibility is not impaired (Rabbitt, 1966; Pichora-Fuller et al., 1995; Amichetti et al., 2013). Other studies have used brain imaging and found that alpha power, an indication of WM load, increased as a function of speech intelligibility (Obleser et al., 2012) and degree of hearing loss of listeners (Petersen et al., 2015). Importantly, these studies established increased power in alpha oscillations during the retention phase of a memory test, suggesting that retaining degraded speech in WM is more effortful than clear speech, even when overall intelligibility is high.

Several studies have established a correlation between tests of verbal WM, typically assessed through the reading-span test (see Daneman and Carpenter, 1980), and performance on SUN tests. The problem with such studies is that no direct causation can be established as performance on both tests may be influenced by a third variable. Specifically, it has been shown that short-term memory (STM) for words is not independent of LTM representations of those words; both word frequency and phonotactic probability influence serial recall of words (Hulme et al., 1991; Hulme et al., 1997; Gathercole et al., 1999). At the same time, SUN is dependent on these factors as described above. Thus the quality of phonological representations in LTM, which is dependent on language experience, may influence both verbal WM and SUN. Therefore, studies that assess the correlation between verbal WM and SUN need to control for language experience to ensure that the correlation is not confounded by this third factor. Two recent studies found that verbal WM was no longer a significant predictor of SUN in a second language when proficiency in that language was controlled for (Kilman et al., 2014; Sörqvist et al., 2014).

Other executive functions next to WM may be recruited during SUN. When individuals follow a conversation in background noise, they have to selectively attend to one speaker and ignore other sounds or speakers (e.g., Mesgarani and Chang, 2012; Wild et al., 2012). In addition, during word recognition, words that are semantically and acoustically related to the target words also become active and inhibiting these competitors may require executive functions (Sommers and Danielson, 1999; Lash et al., 2013). Two recent studies assessed the relationship between individual differences in attention and SUN. Anderson et al. (2013) used structural equation modeling and found that a latent variable consisting of auditory attention, auditory STM, and auditory WM explained a large amount of variance in SUN. However, the contribution of auditory attention was only small compared to the memory measures, which suggests that in this specific study the role of auditory attention was limited. The second study comes from Tamati et al. (2013), who found that individuals who performed high and low on a SUN test did not differ in their performance on a color-Stroop test. This last finding might suggest that auditory attention is more important in SUN than more general attention that is measured by the Stroop test.

The purpose of the present study was to find individual differences that would predict SUN. Based on the ELU model, it was hypothesized that language experience, measured through vocabulary knowledge, verbal WM, and auditory attention would predict SUN. It was further hypothesized that differences between monolingual and bilingual participants would mostly be attributable to differences in language experience. To test this hypothesis, word frequency of to be recognized words was manipulated.

## Materials and Methods

### Participants

The study included 53 monolingual and 48 bilingual participants. The inclusion criteria for monolinguals were that they did not learn a second language before the age of 10. Some monolinguals had learned a second language in foreign language classes in school but they were not fluent in their second language and had not spent more than a short vacation in a non-English speaking country. Bilinguals had to have learned Spanish from birth and English before the age of 8^1^. In addition, participants had to be between 18 and 35 years old. Six additional monolinguals and five additional bilinguals were tested but they were not included in the final sample because they did not meet the definition of monolingual (5), early bilingual (4), or were too old (1) or too young (1) to be included in the study. Detailed participant information can be seen in **Table 1**. The study was approved by the local Institutional Review Board and all subjects gave their written informed consent to participate.

**Table 1:** Participant characteristics divided by landguage group.

	Monolingual	Bilingual
Age in years	20.6 (2.4)	20.8 (2.8)
Number of males	18 (34%)	16 (33%)
Years of formal education	14.9 (1.6)	14.4 (1.4)
Primary caregivers education level:		
-Less than high school	0%	40%
-High school	11%	46%
-Some college	30%	8%
-College	32%	4%
-Some Graduate school	4%	0%
-Graduate school	23%	2%
Self-rated hearing ability (out of 10)	8.6 (1.0)	8.6 (1.1)
Years of musical experience	4.7	1.0
Verbal ability *W*-score	533.2 (8.9)	515.6 (11.4)
Verbal ability standard score	105 (7.7)	90 (8.8)
Picture vocabulary *W*-score	537.1 (11.0)	516.1 (13.5)
Picture vocabulary standard score	101 (7.6)	86 (8.4)
Verbal analogies W-score	529.5 (9.2)	515.3 (11.8)
Verbal analogies standard score	109 (7.3)	98 (9.0)
Verbal ability *W*-score – Spanish	-	503.0 (11.9)
Verbal ability standard-score – Spanish	-	81 (9.3)
Picture vocabulary *W*-score – Spanish	-	500.8 (11.8)
Picture vocabulary standard score – Spanish	-	77 (7.9)
Verbal analogies *W*-score – Spanish	-	505.3 (14.2)
Verbal analogies standard score –Spanish	-	90 (10.8)
Age of acquisition: English	-	4.4 (2.5)
Age of acquisition: Spanish	-	0
Age of arrival in USA	-	1.3 (2.8)
Current amount of time spent		
Listening to English	-	64.6% (18.4)
Speaking English	-	65.5% (17.4)
Reading English	-	81.3% (16.7)

*Years of musical experience: participants were asked if they have ever played a musical instrument or sung in a choir or band and for how many years. See text for an explanation of the different language measures. W-scores are based on an arbitrary equal-interval scale. Standard scores have a population mean of 100 and a standard deviation of 15. Values in parentheses are standard deviations*.

### Experimental Design

#### Background Questionnaire

Participants’ background information was collected with a questionnaire created for this study, administered by the experimenter. The instrument was loosely based on Marian et al. (2007) but included additional information about parental education and use of English and bilingual participants’ use of English and Spanish during their childhood and adolescence. It took about 6–10 min to administer.

#### Speech Understanding in Noise

Materials for the SUN test were taken from the revised Speech Perception in Noise test (SPIN; Bilger et al., 1984), which was obtained as a digitized recording. The test consists of 200 target words and each word is recorded in a predictive and unpredictive context. For example, the word *coast* could be preceded by *Ms. Brown might consider the coast* (low predictability) or by *The boat sailed along the coast* (high predictability). The original SPIN recordings were obtained on CD from the Department of Speech and Hearing Science from the University of Illinois Urbana-Champaign. The sound file was edited so that each sentence was saved in a separate file. For the background babble, a short sequence from the original babble track (12-talker babble) was chosen and mixed with each sentence in Praat (Boersma and Weenink, 2014) at two different speech-to-noise ratios (SNRs; -2 dB and 3 dB). These SNRs were chosen based on a pilot experiment. The sound intensity of the sentence was held constant and so the intensity of the babble differed for the two SNRs.

In the present study, 128 sentences from the test were chosen and divided into four lists of 32 words^2^. Words in each list were matched on word frequency, phonotactic probability, and on neighborhood density. Information about lexical variables was taken from different sources. Information about lexical frequency was taken from Brysbaert and New (2009). These norms are based on a large corpus created from subtitles of American movies and TV shows. The mean log10 word frequency of the stimuli used in the present study was 2.70 (*SD* = 0.44) and the mean frequency per million was 15.92 (*SD* = 16.46). Information about phonotactic probability came from Vitevitch and Luce (2004). This database provides the summed probabilities of each phoneme in a word and the summed probability of each biphone. The number of neighbors of a word were calculated based on the English Lexicon Project (Balota et al., 2007). The correlation between biphone probability and log-frequency was *r* = 0.16 and the correlation between log-frequency and neighborhood density was *r* = 0.16.

Each participant heard the first half at 3 dB SNR and the second half at -2 dB. Within each SNR, half of all words were played in a predictive context and the other half in an unpredictive context in a randomized order. Across all participants, each word was administered in all four conditions in a Latin-square design. After each sentence, the participant was prompted to type the last word of the sentence. The next trial started when a participant pressed ENTER. Before the actual experiment, 10 sentences were administered at a SNR of 8 dB to ensure that participants had understood the task. Participants were also told to check the word they typed on the screen for any spelling errors before going to the next trial. This test was administered in Eprime 2.0 (Psychology Software Tools, Sharpsburg, PA, USA).

#### Working Memory

The WM test used for this study comes from the US National Health Institute’s (NIH) so called Toolbox^3^. The NIH toolbox is a collection of different tests in the areas of cognition, emotion, motor function, and sensation. All tests are available freely and are administered online. In the WM test, participants see pictures and their labels and hear their names. The set-size differs from two to seven pictures. Pictures are either animals or food items. After each set of pictures, participants are asked to repeat what they just saw in size order from smallest to biggest. For example, if they saw a bear, a duck, and an elephant, they would say duck, bear, and elephant. To establish the size order, participants have to pay attention to the size of the object on the screen but in most cases, the relative proportions on the screen corresponded to real life. The test has two parts. In the first part, sets consist only of animals or only of food items. In the second part, sets consist of animals and food and participants are asked to repeat the food first from smallest to biggest and then the animals from smallest to biggest. Both parts start with two practice sets to ensure that participants understood the directions. If they make a mistake in either practice set, the instructions are repeated and the set is administered again. After the practice items, the test starts with a set size of two. If a participant correctly repeats all pictures, the set size of the next trial increases by one. If the participant makes an error, another set of the same size but different items is administered. Testing stops when a participant cannot correctly repeat two sets in a row or when the last set is administered. Responses were recorded on a paper sheet and a score for each participant was calculated by counting the total number of items of all correctly repeated sets. Thus the total score for each part is 27 (2+3+4+5+6+7) and the total possible score is 54. This test was only administered in English.

Recently, the reliability of the test was established (Tulsky et al., 2014). The test–retest interclass correlation coefficient was 0.77. The test also correlated with other established WM tests (*r* = 0.57) and tests of executive function (*r* = 0.43 -0.58). The correlation with a test of receptive vocabulary, on the other hand, was low (*r* = 0.24).

#### Verbal Ability

Verbal ability was assessed with the Woodcock-Muñoz Language Survey – Revised (WMLS-R; Woodcock et al., 2005), which is a norm-referenced, standardized test of English and Spanish. Both versions were normed on a large sample of speakers in the US and Latin America in the case of the Spanish version. The raw-score on the test can be transformed into a standard score with a population mean of 100 and a standard deviation of 15 through software that is provided with the test (Schrank and Woodcock, 2005). In addition, scores can be expressed as W-scores, which are based on an equal interval scale and are therefore suitable for statistical analyses and group comparisons. Unlike standard scores, *W*-scores are not corrected for participant age at testing.

The WMLS-R consists of seven tests, two of which were administered in the present study. The first one is called Picture-Vocabulary test. Participants are shown pictures in sets of six and are asked to name them one by one as the experimenter asks them “What is this” while pointing at a picture. The second test administered is called Verbal Analogies. Participants are asked to solve “riddles” such as *In is to out as down is to …?* Scores from both tests can be combined into a single score with the provided software, which the test developers call Oral Language Ability (henceforth verbal ability). This score correlates highly with the cluster score that is based on all tests of the WMLS-R (*r* = 0.9). The standard error of the mean for all tests is between 5.55 and 5.93 and the internal consistency reliability coefficients were around *r*_11_ = 0.9 (Alvarado and Woodcock, 2005).

#### Auditory Attention

The auditory attention test was adapted from Zhang et al. (2012). In this test, participants have to decide whether two tones were played to the same ear or different ears. What makes this test challenging is that the frequency of the two tones is sometimes the same and sometimes different. Because participants are only supposed to respond based on the location of the tones, response conflict arises on trials in which the location is different but the frequency the same or the location the same and the frequency different. The manipulation of frequency and location results in four conditions, same-frequency same-location (SFSL), same-frequency different-location (SFDL), different-frequency same-location (DFSL), different-frequency different-location (DFDL). The original test also has a second part where frequency is the task-relevant dimension and location is the irrelevant dimension that has to be ignored. However, only the first condition was used in the present study to reduce the time needed to administer the test.

Three different measures can be derived from the test, baseline RT, involuntary orientation, and conflict resolution. Baseline RT is the mean RT in the SFSL condition. In Zhang et al. (2012), baseline RT correlated with the RTs in a separate test that did not involve response conflict and therefore the authors suggested that this measure reflects information processing speed. Involuntary attention can be calculated by subtracting RTs on trials with the same frequency from those of different frequency [(DFDL+DFSL) –(SFSL+SFDL)]. Conflict resolution can be calculated by subtracting the mean RTs on trials where location and frequency were both different or both the same (no response conflict) from those where they were different [(SFSL+DFDL)–(SFDL+DFSL)]. Preliminary correlational analyses (see Supplementary Materials) with each of these three measures and overall accuracy on the SUN test showed that only processing speed correlated significantly with SUN accuracy and so only this variable was used in the analyses reported below.

The tones for this test were created in Praat (Boersma and Weenink, 2014) as pure tones with a length of 100 ms. The frequency ranged between 500 and 1400 Hz in 100 Hz intervals, which resulted in ten different sound files. For different-frequency trials, the second tone was randomly chosen. There were a total of 96 experimental trials, 24 trials in each condition. The experiment was programmed in E-Prime.

### Procedure

All participants completed all tests in the following order: consent form, background questionnaire, attention test, Words-in-Noise test (Wilson et al., 2007, not reported here), SUN test, verbal ability, WM test, and a consonant perception test (not reported here). Bilingual participants then also completed the verbal ability and the Words-in-Noise test in Spanish.

### Analysis

Incorrect responses on the SUN test were manually checked for any spelling mistakes. A misspelled word was counted as correct in the following cases: letter transposing (e.g., thief for thief), wrong letter when the correct letter was adjacent to it on the keyboard and the resulting word was not a word in English (e.g., ahore for shore), when a letter was missing and the resulting word was not a word in English, or when the answer was a homophone of the target word, regardless of whether the typed word was a real English word (e.g., gyn or jin for gin). In total, 286 (2.2%) instances were corrected in this way, which is comparable to 2.5% in Luce and Pisoni (1998) who used a similar procedure.

For the analysis, mixed-effects regression models were run in R (R Core Team, 2015) using the *lme4* package (Bates et al., 2015). *P*-values were calculated using the Anova function in the *car* package (Fox and Weisberg, 2011) using the type II sums of squares method. Subjects and items were entered as random effects.

## Results

**FIGURE 1:**
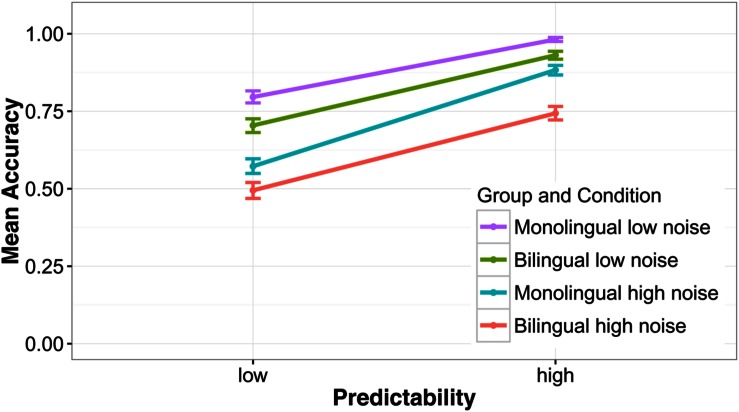
**Mean accuracy on the speech understanding in noise test**. Sentences were presented at two noise levels (low/high) and the predictability of the target word was either high or low. Error bars show the 95% confidence interval.

**FIGURE 2:**
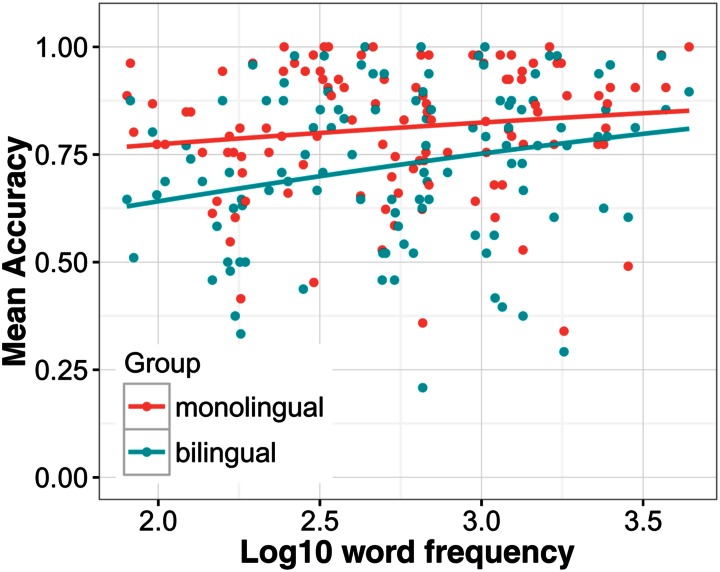
**The effect of word frequency on speech understanding in noise accuracy**. Regression lines show the best fit. Each dot represents the mean accuracy for a certain word in each group.

First a model was run with four predictor variables to analyze group-level effects, language group (bilingual/monolingual), predictability (low/high), noise level (low/high), and word frequency (see the Supplementary Materials for model specifications). The results showed that words in low noise (*M* = 85.5%, *SD* = 35.2) were recognized with higher accuracy than words in high noise [*M* = 67.6%, *SD* = 46.8; χ^2^(1) = 712.4, *p* < 0.001], and words in a predictive context (*M* = 88.7%, *SD* = 31.6) better than words in an unpredictive context [*M* = 64.4%, *SD* = 47.9; χ^2^(1) = 1059.3, *p* < 0.001]. The difference between a low and a highly predictive context was 28.2% when noise was high and 20.5% when noise was low and this interaction was significant [χ^2^(1) = 30.7, *p* < 0.001]. Monolinguals recognized words more accurately (*M* = 80.8%, *SD* = 39.4) than bilinguals [*M* = 71.8%, *SD* = 45.0; χ^2^(1) = 76.7, *p* < 0.001]. When noise was low, the difference between monolinguals and bilinguals was smaller [*M*_Δ_ = 7.1 percentage points (pp)] than when noise was high (*M*_Δ_ = 10.9 pp) but this interaction did not reach significance [*χ*^2^(1) = 3.19, *p* = 0.074]. The effect of predictability was only slightly larger for monolinguals (*M*_Δ_ = 24.8 pp) than bilinguals (*M*_Δ_ = 23.8 pp). Nevertheless, the interaction between predictability and language group was significant [χ^2^(1) = 47.56, *p* < 0.001]. As can be seen in **Figure 1**, this interaction was likely caused by the fact that monolinguals benefitted more from a predictive context compared to bilinguals when noise was high. In the high noise condition, the benefit for monolinguals was *M*_Δ_ = 31.06 pp and *M*_Δ_ = 24.87 pp for bilinguals. The main effect of frequency was significant [χ^2^(1) = 6.00, *p* = 0.014], showing that high frequency words were recognized with greater accuracy than low frequency words. The interaction between frequency and language group was also significant [χ^2^(1) = 5.65, *p* = 0.017]. **Figure 2** suggests that this interaction was driven by the steeper slope of the frequency effect in the bilingual group compared to the monolingual group.

The following variables were added to the analysis to investigate the effect of individual differences: verbal ability, WM, and processing speed. All continuous variables were centered around the mean. The mean values for each variable can be seen in **Table 2**. WM and verbal ability were highly correlated [*r*(99) = 0.527, *p* < 0.001] and WM and processing speed were moderately correlated [*r*(99) = 0.229, *p* = 0.021]. Processing speed and verbal ability were not correlated [*r*(99) = 0.034, *p* = 0.737; see the Supplementary Materials for a detailed correlation matrix].

**Table 2:** Mean values for the individual differences variables.

	Verbal ability	Working memory	Processing speed
Monolingual	533.2 W (8.9)	37.6 (8.0)	680 ms (125)
Bilingual	515.6 W (11.4)	32.4 (7.9)	702 ms (139)
Total sample	524.8 W (13.4)	35.2 (8.3)	690 ms (132)

*W-scores are based on an arbitrary equal-interval scale. See text for an explanation of working memory scores. Processing speed is the baseline mean response time on the attention test. Standard deviations are shown in parentheses*.

A model was built with the same variables as above, that is, language group, word frequency, noise level, and predictability, plus the individual difference variables. Besides the main effects, only the significant interactions are reported here. The full model can be seen in the Supplementary Materials.

**FIGURE 3:**
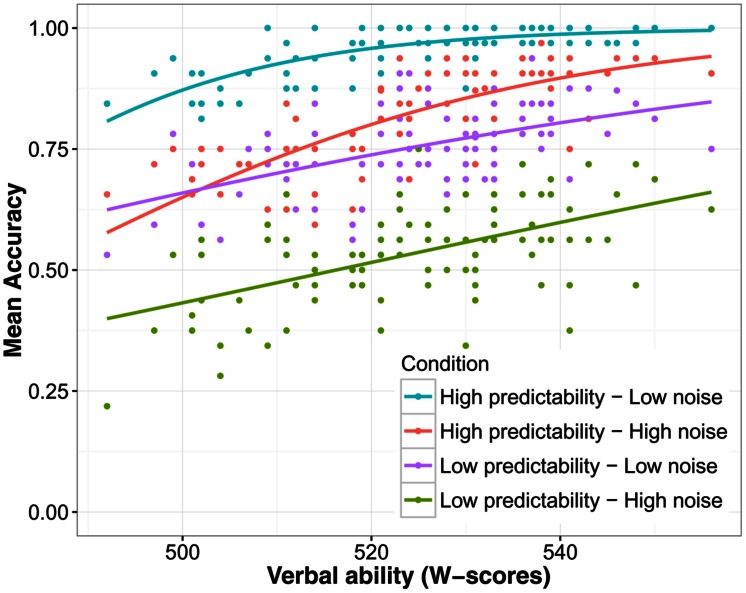
**The effect of verbal ability on speech understanding in noise accuracy**. *W*-scores are on an arbitrary equal-interval scale. Each dot in each condition represents one participant.

**FIGURE 4:**
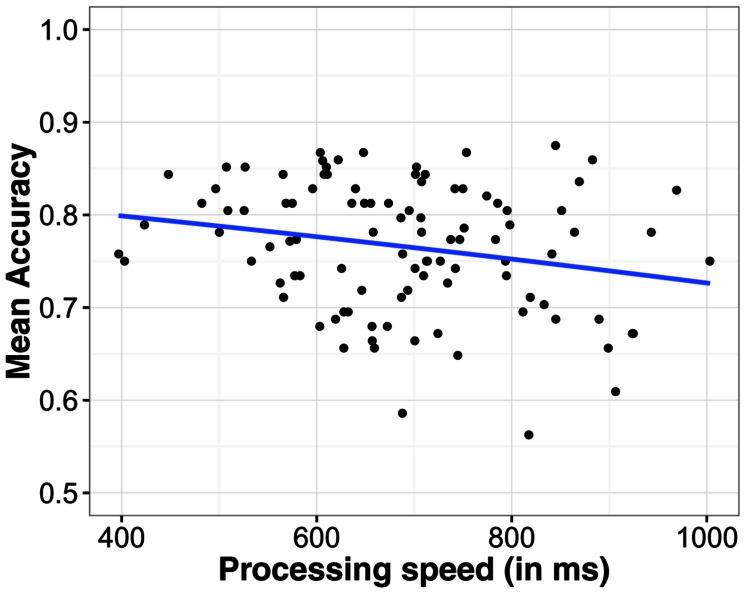
**The effect of processing speed on speech understanding in noise accuracy**. Processing speed was the baseline measure on the attention test. Note the reduced range of the *y*-axis to highlight the effect.

**FIGURE 5:**
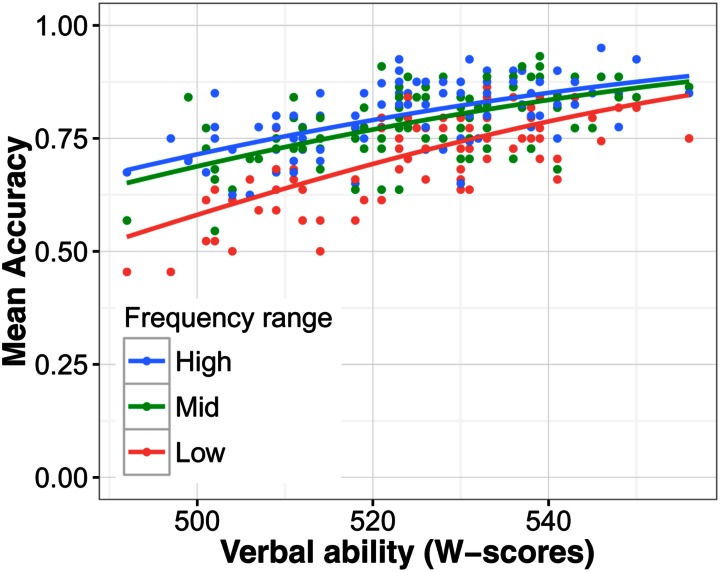
**The effects of oral language ability and word frequency on speech understanding in noise accuracy**. To show the effect of word frequency, the continuous variable was split into a factor with three levels, high, mid, and low frequency.

The main effects of language group, noise level, and predictability were highly significant as before (all χ^2^ > 10, *p*s < 0.001). Furthermore, main effects of verbal ability [χ^2^(1) = 44.51, *p* < 0.001] and processing speed [χ^2^(1) = 5.87, *p* = 0.015] were significant, showing that higher verbal ability and faster processing speed (lower RTs) were associated with higher accuracy on the SUN test. This can be seen in **Figures 3** and **4** respectively. The interaction between verbal ability and predictability was significant [χ^2^(1) = 53.10, *p* < 0.001]. As **Figure 3** shows, participants with higher verbal ability benefitted more from a predictive context compared to those with lower verbal ability. The interaction between word frequency and verbal ability was also significant [χ^2^(1) = 5.13, *p* = 0.024]. This interaction can best be interpreted using **Figure 5**. The difference in accuracy between listeners with high and low verbal ability was most pronounced for low frequency words. WM was not a significant predictor of SUN accuracy [χ^2^(1) < 0.01, *p* = 0.978], likely because of its high correlation with verbal ability (when verbal ability was taken out of the model, WM became a significant predictor; see Supplementary Materials). These analyses show that verbal ability was a powerful predictor of SUN accuracy. Expressed as a odds-ratio, compared to someone with average verbal ability, someone with verbal ability 1 SD above the mean was 2.14 times more likely to recognize a target word. Compared to verbal ability, the effect of processing speed was much smaller. Compared to someone with mean processing speed, someone 1 SD below the mean was 1.09 times more likely to recognize a target word.

To check whether the effect of verbal ability was true for both groups or was simply driven by group differences, follow-up analyses were run for each group separately. The main effect of verbal ability and the interaction with predictability were highly significant in both groups (all χ^2^ > 15, *p*s < 0.001) but the interaction with frequency was no longer significant (both χ^2^ < 1). The main effect of frequency was significant in the bilingual group [χ^2^(1) = 8.61, *p* = 0.003] but not in the monolingual group [χ^2^(1) = 3.27, *p* = 0.071]. Furthermore, the effect of processing speed did not reach significance in either group (*p*s = 0.058 and 0.129 for the bilingual and monolingual group, respectively). This may have been due to insufficient power in these smaller samples.

**FIGURE 6:**
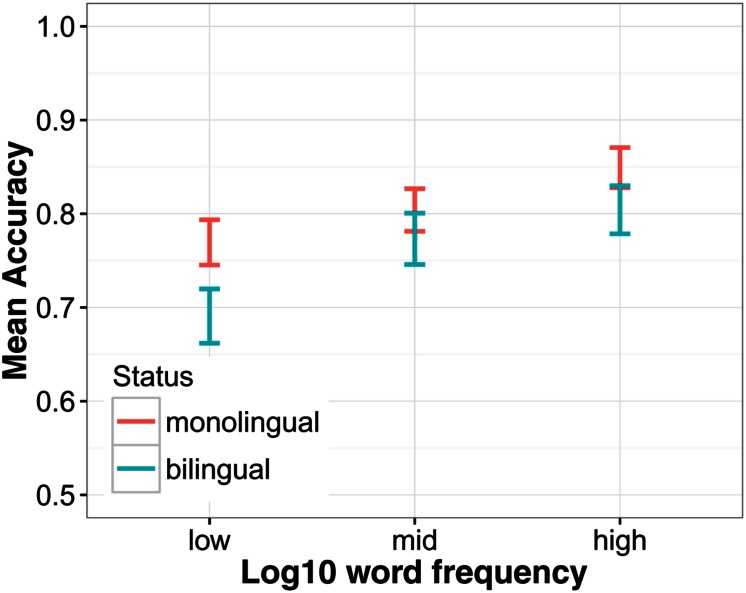
**The effect of word frequency in a subsample of monolinguals and bilinguals matched on vocabulary knowledge**. Word frequency was split into a factor with three levels, high, mid, and low frequency, to highlight the effect. Also note the limited range of the *y*-axis.

The analyses so far suggest that verbal ability had an effect on SUN in both the monolingual and the bilingual group. Yet, even when verbal ability was controlled for, language group was still a significant predictor. To investigate further what the added difficulty for bilinguals might be, two subgroups were formed from each group, respectively, that were closely matched on their vocabulary score^4^ by randomly selecting participants from each group with a similar score (see **Table 3**). A *t*-test confirmed that the difference in vocabulary scores between these subgroups was not significantly different [*t*(44) = 0.63, *p* = 0.534]. The mean group difference in SUN accuracy in this subsample was *M*_Δ_ = 5.1 pp, which is smaller than in the total sample (*M*_Δ_ = 9.0 pp). Yet this difference was still statistically significant [χ^2^(1) = 15.35, *p* < 0.001]. The interaction between word frequency and language group was not significant [χ^2^(1) = 2.02, *p* = 0.155] but **Figure 6** suggests that it was especially the low frequency words that were more difficult for bilinguals. Also the language group by predictability interaction was still significant in this subsample [χ^2^(1) = 4.07, *p* = 0.044], suggesting that differences in language proficiency alone cannot explain this interaction.

**Table 3:** A subsample from each group matched on language proficiency.

	*n*	PV score	VA score	SUN accuracy
Monolingual	24	529.8 W (7.8)	521.3 W (11.5)	80.5% (39.7)
Bilingual	22	528.3 W (8.1)	526.8 W (9.5)	75.4% (43.1)

*A subsample from each group was randomly chosen and matched on their picture vocabulary score. PV = picture vocabulary. VA = verbal ability. SUN = speech understanding in noise. Standard deviations are shown in parentheses*.

## Discussion

The results confirmed previous studies by showing that noise had a disruptive effect on speech understanding whereas a predictive context was facilitative. The effect of a predictive context was stronger when noise was high compared to when it was low and monolinguals benefitted more from a predictive context than bilinguals. Word frequency had an effect on recognition accuracy, high frequency words were recognized with greater accuracy than low frequency words. However, in follow-up analyses, this effect was only marginally significant in the monolingual group, while it remained significant in the bilingual group. Next, an analysis of the effect of individual differences in verbal ability, WM, and attention was conducted. The effect of verbal ability was highly significant in both groups, as was the interaction between verbal ability and predictive context, showing that individuals with higher verbal ability recognized more words in general and also benefitted more from a predictive context. The effect of WM was not significant, likely because of its shared variance with verbal ability. The effect of processing speed was significant when both groups were analyzed together but did not reach significance when each group was analyzed separately. Finally, two subsamples from each group that were matched on their vocabulary scores were compared. This analysis showed that group differences were reduced when subjects were matched on verbal ability but the differences were still statistically significant, suggesting that differences in verbal ability cannot completely explain the bilingual disadvantage in SUN.

As in previous studies (e.g., Mayo et al., 1997; Meador et al., 2000; Rogers et al., 2006; Bradlow and Alexander, 2007; Shi, 2009, 2010; Van Engen, 2010), the bilingual speakers recognized fewer words on average than the monolingual speakers. However, the effect was additive rather multiplicative, meaning there was no interaction between noise level and group. This is in line with Rogers et al. (2006). Yet, other studies found an interaction (Mayo et al., 1997; Shi, 2010; Tabri et al., 2011). The reason may be that in the present study only two noise levels were tested. Another reason may be that the bilinguals in the present study learned English early and had grown up in an English-speaking environment. They were thus more proficient than many of the second language speakers tested in previous research.

An improvement to many previous studies that compared monolingual to early bilingual listeners (e.g., Mayo et al., 1997; Shi and Sánchez, 2010) was the inclusion of a larger sample. Thus there is more robust evidence that even early bilinguals have greater difficulties recognizing words in noise. Previous research also established that more exposure, a younger age of acquisition, and greater proficiency in the target language is positively associated with SUN (Meador et al., 2000; Shi, 2009, 2012; Rimikis et al., 2013; Kilman et al., 2014). The present study sought to contribute to the current discussion of bilingual SUN by not only showing the existence of a so-called *bilingual disadvantage* and which factors contribute to it but also to find possible explanations for this disadvantage. In this respect, an improvement to previous research was that monolingual and bilingual participants were tested with the same standardized language test. A standardized test is not only important to make results comparable across studies but also to be able to compare the samples of monolinguals and bilinguals within a study. This is important to note because the present study found that verbal ability was associated with SUN in both groups. Since bilinguals often have a smaller vocabulary in each of their languages compared to monolinguals (e.g., Portocarrero et al., 2007; Bialystok and Luk, 2012; Gasquoine and Dayanira Gonzales, 2012), one reason for the bilingual disadvantage for SUN in previous studies may be that groups simply differed in verbal ability. This assumption was confirmed when two subsamples were compared that were matched on vocabulary size. Compared to the total sample, the difference went down from 9.0 to 5.1 pp, which is a decrease of 43%. At the same time, differences in language proficiency cannot be the only explanation because even these two subsamples matched on proficiency were still significantly different in SUN accuracy.

Word frequency may be a second, albeit related, explanation for the bilingual disadvantage in SUN. All participants recognized high frequency words with higher accuracy than low frequency words. As described in the introduction, exemplar models of speech perception assume that each encounter with a word leaves a trace in memory and that words that are encountered frequently are represented in memory with more phonetic detail. The more a word is encountered in different contexts, spoken by different speakers, the more robust its recognition will be under suboptimal listening conditions. Because on average bilingual speakers have not had as much exposure to each of their languages compared to a monolingual speaker, all words are encountered less often (c.f. Gollan et al., 2008) and disproportionately less so low frequency words (Kuperman and Van Dyke, 2013). This can explain the interaction between group and word frequency, which showed that the bilinguals as a group recognized especially low frequency words with lower accuracy than monolinguals. This explanation suggests that the bilingual disadvantage stems from their reduced exposure to each of their languages. Thus we would expect the same to be true for monolinguals who, for various reasons, have not had as much exposure to lower frequency words. For example, Tamati et al. (2013) tested a large sample of native English speakers on a SUN test and also had subjects rate their subjective familiarity with certain words. They found that those who performed well on the test reported to be more familiar with low frequency words than those who performed not so well on the test. Assuming that familiarity is closely related to the frequency of encounter with a word, their study and the present one suggest that subjective word frequency is an important factor influencing individual differences in SUN.

Both explanations for individual differences in SUN, verbal ability and word frequency, are related because both depend on language experience. Someone who is exposed to language in many different contexts is more likely to learn the meaning of more words compared to someone with more limited exposure and, at the same time, they will encounter words of lower frequency more often. How, then, can we explain that the two subsamples that were matched on verbal ability still performed significantly different on the SUN test? It may be that for the bilinguals, vocabulary knowledge overestimated their actual exposure to English. Even though they knew the meaning of a less common word, they may not have encountered that word as often as a monolingual speaker. Also, assuming that a bilingual speaker hears English in school and Spanish outside of school, they will hear each language not only less often but also from a more limited number of speakers. These may be factors that determine the quality of phonological representations (Gollan et al., 2014; Schmidtke, 2015) and thus SUN. Suggestive of this explanation is that, as in the whole sample, the largest difference between these two subsamples was in the low frequency range (see **Figure 6**), although the interaction between group and frequency did not reach significance. In this respect it is interesting that the size of the frequency effect changed as a function of proficiency. The effect was most pronounced for participants at the lower end of the proficiency range. In the matched subsamples, however, the proficiency range was smaller and this may be why the interaction was no longer significant.

While the present hypothesis for the bilingual disadvantage was based on exemplar models, the data do not necessarily contradict the predictions of models that assume an abstract level of representation of words. For example, TRACE (McClelland and Elman, 1986) assumes three levels of representation, a feature level, a phoneme level, and a word level, with each level of representation being more abstract. Frequency effects can be modeled by adjusting the resting-activation levels of words so that words with high resting levels require less activation from the speech signal, which results in earlier selection compared to words with low resting-activation levels (Dahan et al., 2001). A noisy signal could result in fewer features that receive activation so that words with a low resting-activation level do not receive sufficient activation to pass the threshold necessary for selection. Proponents of a mental lexicon with abstract representations of words can explain differences between native and non-native speech perception by assuming differences at a perceptual level. Because categorical speech perception develops very early in life (Kuhl, 2004), even an early learned second language will be perceived through the phonemic inventory of the first language (e.g., Sebastián-Gallés and Soto-Faraco, 1999), which will result in non-native-like phonological representations in the mental lexicon (Pallier et al., 2001). However, the two models do not have to stand in opposition to each other and more recently researchers have developed hybrid models that include aspects of exemplar and abstract models to be able to explain the whole range of phenomena (e.g., Goldinger, 2007; Ernestus, 2014; Kleinschmidt and Jaeger, 2015; Pierrehumbert, 2016). This being said, exemplar models provide a more elegant solution to explain the present results. Differences in the quality of mental representations of words between and within speakers are a fundamental part of exemplar-based models and so they can readily explain individual differences in word recognition. Abstract models, on the other hand, have to assume additional mechanisms to be able to explain individual differences.

Exemplar-based models may also be useful to explain the finding that individual differences in WM capacity were not a significant predictor of SUN when controlling for language ability. A verbal WM test was included in the current study because of the ELU’s prediction that individuals with a larger WM capacity would recognize words in noise with less effort and thus be more accurate. The test required individuals to remember items in different set sizes and to mentally manipulate the order of the items according to their size. Because of these storage and processing components, the test is believed to tap into WM. Individuals who can correctly recall more sets are assumed to have a larger WM capacity. The items were common animals and food items such as *mouse*, *pig*, and *banana* that all participants were likely very familiar with. It was therefore surprising that the test correlated highly with the language test (*r* = 0.5). Exemplar-based models can explain this finding because they assume that not only one representation is activated at the time of encoding but all exemplars of a word. If a word is represented by many exemplars then it is more likely that a memory trace is still active in LTM at the time of retrieval. Related to this explanation is also the finding that items stored in WM are not independent from LTM representations (e.g., Hulme et al., 1997; Acheson et al., 2011). Additionally, in individuals with larger mental lexicons the phonological representations of words may be overall more precise, which may reduce the spread of activation to similar sounding words and therefore prevent interference during rehearsal (cf. Cowan et al., 2005). However, although WM was not a significant predictor of SUN accuracy in the present sample, this does not necessarily imply that individual differences in WM are not important for SUN. The participants here were all young adults and a more diverse sample in terms of age may be needed to find an effect of verbal WM above and beyond verbal ability. For example, Parbery-Clark et al. (2011) found a correlation between auditory WM and SUN ability even when controlling for vocabulary knowledge in a sample of older listeners. But the present results may further inform the ELU in that the quality of lexical representations in LTM and capacity limits of WM are not independent constructs. This view would be more akin to the model of WM developed by Cowan et al. (2005) and Cowan (2008) rather than to a limited capacity system for temporary storage of items as it is currently defined in the ELU (Rönnberg et al., 2013, p. 2). The present results also have implications for future research. Researchers interested in the relationship between SUN and cognition should always also include a proficiency test that measures vocabulary knowledge in their test batteries when they administer a verbal WM test. Otherwise correlations may be attributed to WM (or some other covariate) when in fact language experience is the underlying factor. However, the type of verbal ability test used may also lead to differing results, since an effect of verbal ability is not always found (e.g., Benichov et al., 2012). In the same way, in the norming study of the WM used here the authors found a much weaker correlation between WM and receptive vocabulary (*r* = 0.24; Tulsky et al., 2014).

The next finding that merits discussion is the effect of a predictive context. Previous research found that bilingual and second language speakers do not benefit as much from a predictive context as monolinguals under certain circumstances (Mayo et al., 1997; Bradlow and Alexander, 2007; Shi, 2010). However, the present results suggest that individual differences in the effective use of context also exist between monolinguals and that verbal ability is the mediating factor. This would again suggest that differences between monolinguals and bilinguals might emerge because of differences in verbal ability (see above). As a result, the less effective use of context cues attributed to bilingualism is not a bilingual disadvantage *per se* but may be a result of reduced language experience (cf. Newman et al., 2012). But what is the relationship between verbal ability and the effective use of context cues? One explanation is that individuals with lower verbal ability generally understood fewer words and so if they missed words in the preceding context of the target words, they were not able to form any predictions. Another explanation may be the relationship between verbal ability and WM. In order to make predictions about the target word, subjects need to maintain preceding words in WM. This process might take up more resources depending on the ease with which phonological representations are retrieved and maintained in WM. A third explanation may be the association strength between words (Spence and Owens, 1990). One example sentence from the SUN test is *the ship sailed along the coast*. Here, *ship* and *sailed* may be used to predict the target word *coast*. If individuals with larger vocabularies have more language experience overall, then they have likely heard words such as ship and coast more often in the same context and thus there is a stronger association of ship and coast compared to an individual with less language experience (c.f. Nation and Snowling, 1999).

Given the findings discussed so far, a frequency-based explanation of differences between monolinguals and bilinguals seems to be the most powerful because it cannot only account for group differences but also differences between individual participants. Furthermore, a frequency-based account can give a united explanation of the language-related effects such as language proficiency, word frequency, predictive context, and the null-effect of verbal WM. The last variable to be discussed, attention, stood out in this respect because it was not language related. The attention test was included in the study to give a more complete picture of individual differences in SUN, as recent studies have pointed to the potential role of non-linguistic factors in language comprehension and especially SUN (e.g., Anderson et al., 2013; Fedorenko, 2014).

The attention test based on Zhang et al. (2012) provided three different variables but no prediction was made as to which variable would be associated with SUN. In the analysis, only processing speed was used because it provided the most robust correlations with the SUN test of the three variables. The results showed a small but significant effect of processing speed on SUN accuracy. The reason why this effect was small might be that there was not enough variance in the data for a stronger effect to emerge. As with WM, processing speed may become more important as a factor in older populations. The general speed of information processing slows down in older adults (Salthouse, 1996), which may explain why cognitive factors are sometimes a better predictor of SUN than hearing acuity (Wingfield, 1996; Benichov et al., 2012). However, further studies are needed to confirm or disconfirm that processing speed is indeed a better predictor of SUN than the conflict resolution or involuntary attention components of the test.

One practical implication of the study for hearing testing is that word frequency needs to be taken into account. One possibility is to only use high frequency words when testing patients to avoid a possible confound. On the other hand, it may be useful to test high and low frequency words and to have norms for each set. If a patient fares especially poor for the low frequency words then this might be an indication for the practitioner that part of the patient’s hearing difficulties may stem from factors unrelated to hearing acuity.

Some limitations of the present study that qualify the results should be addressed. Inherent to the design of the study, no inferences about causation can be made. The results suggest that a larger vocabulary is associated with better SUN but the nature of this relationship requires further investigations. Here the assumption was made that exposure frequency is the mediating variable but vocabulary size could also have a direct influence on word recognition. Alternatively, though less likely, people with better SUN ability may be better able to pick up new words through listening and therefore have larger vocabularies. Another limitation is that only one WM and one attention test were used. Future studies would benefit from the use of multiple tests for each construct, which, along with a larger sample size, would allow more sophisticated statistics such as structural equation modeling. Finally, the two samples did not only differ in language status (monolingual vs. bilingual) but also in the age of acquisition (AoA) of the tested language and socio-economic status (SES; assessed by maternal education level). In the present study, additional tests showed that neither variable was a significant predictor of SUN once language proficiency was accounted for but these results may be different in a sample where AoA and SES are not correlated with verbal ability.

## Conclusion

The purpose of the present study was to find factors that would explain individual differences in SUN between listeners, especially between monolingual and bilingual listeners. Previous research had established that bilinguals often performed below monolinguals on SUN tests, even when the bilinguals had learned the second language early in life. The present study confirmed these results but the general conclusion was that differences between groups could largely be explained by frequency effects, which suggests that differences between groups are less categorical than might be assumed based on previous research. Based on the ELU model (Rönnberg et al., 2013), it was hypothesized that listening difficulty arises from mismatches between the speech signal and internal phonological representations. Mismatches can occur because of a poor signal and because of poor phonological representations in LTM. In the current ELU model, the definition of what poor phonological representations are is underspecified and so the ELU was extended to exemplar models of the mental lexicon (e.g., Goldinger, 1996, 1998). These models assume that each encounter with a word leaves an episodic trace in memory. The present study showed that recognition of high frequency words was more robust to noise compared to low frequency words. Exemplar models can explain this finding in that high frequency words are represented in memory with more exemplars and more highly activated exemplars than low frequency words (Pierrehumbert, 2001). Word retrieval of high frequency words is more robust because a new exemplar will more likely be similar to an already stored exemplar when more exemplars of a word exist in memory. Following these assumptions, the premise of the study was that the bilingual disadvantage in SUN is a frequency effect (c.f. Gollan et al., 2008). Because bilinguals are exposed to each of their languages less often than monolinguals, they encounter all words less frequently. Consequently, bilinguals will have fewer stored exemplars in LTM for all words. This will especially affect the recognition of low frequency words as bilinguals will encounter these even more rarely than monolinguals and consequently recognition of these words under noise is expected to be more fragile. In support of this hypothesis, the present study found that differences in SUN between groups were largest for low frequency words. Another consequence of reduced exposure to each language is a smaller vocabulary. As in previous research (Portocarrero et al., 2007; Bialystok and Luk, 2012), bilinguals scored on average below monolinguals on verbal ability test, and higher verbal ability was associated with better performance on the SUN test. Importantly, however, there was a relationship between verbal ability and SUN for both groups, suggesting that some of the group differences might be explained by the overall lower English proficiency of the bilinguals. When two subgroups that were matched on language proficiency were compared, the difference in performance on the SUN test was much smaller (5.1% compared to 9.0%). These results support the hypothesis that differences in SUN between monolinguals and bilinguals are a result of the bilinguals’ reduced exposure to each of their languages as a consequence of being bilingual.

## Conflict of Interest Statement

The author declares that the research was conducted in the absence of any commercial or financial relationships that could be construed as a potential conflict of interest.

## Footnotes

1 Four bilinguals reported to have learned English later than 8 but they were included in the study because they were born in the US and attended school in the US from kindergarten. They reported that they attended a Spanish–English bilingual program but that little English was taught. However, they likely had some exposure to English. Thirty-seven (77%) bilinguals were born in the US. Of the remaining bilinguals, all but five arrived in the US before the age of 6. Four of those immigrated at the age of 7 and one at the age of 13. The latter participant was included because her mother was a native speaker of English and she had learned both English and Spanish from birth and attended a bilingual school.

2 The word *fun* was later dropped from all analyses because its frequency per million of 235 was several SDs away from the mean of 15.9. Thus there were 127 unique items.

3 www.nihtoolbox.org.

4 For matching, only the vocabulary score (i.e., Picture Vocabulary) was compared because in the bilingual group, English verbal reasoning was correlated with Spanish verbal reasoning and so vocabulary is likely a better indicator of English exposure.
