# Supplementary material for: The Bilingual Disadvantage in Speech Understanding in Noise Is Likely a Frequency Effect Related to Reduced Language Exposure
Source: Front Psychol. 2016 May 13;7:678. doi: 10.3389/fpsyg.2016.00678 (PMC4865492; doi:10.3389/fpsyg.2016.00678)
Supplement: Supplementary file 1 [file Data_Sheet_1.PDF]

Supplementary Material

The bilingual disadvantage in speech understanding in noise is likely a frequency effect related to reduced language exposure

Jens Schmidtke*

*** Correspondence:** Jens Schmidtke: schmi474@msu.edu

1 Supplementary Tables

Model 1

Formula
glmer(Accuracy~ Noise*Predictability*Status*Lg10WF + (1|Subject) + (1|Word), family='binomial', control=glmerControl(optimizer="bobyqa")))

Fixed Effects

**Predictor variable**	χ^2	**Df**	***p***
Noise	707.6	1	< .0001
Predictability	1052.4	1	< .0001
Group	76.7	1	< .0001
Lg10WF[1]	6.0	1	.0143
Noise:Predictability	29.1	1	< .0001
Noise:Status	2.7	1	.0982
Predictability:Status	45.2	1	< .0001
Noise:Lg10WF	2.2	1	.1351
Predictability:Lg10WF	0.9	1	.3303
Status:Lg10WF	5.7	1	.0174
Noise:Predictability:Status	0.1	1	.8175
Noise:Predictability:Lg10WF	2.9	1	.0911
Noise:Status:Lg10WF	0.2	1	.6657
Predictability:Status:Lg10WF	0.0	1	.9182
Noise:Predictability:Status:Lg10WF	0.9	1	.3423

Random Effects

**Variable**	**Variance**	**SD**
Subjects (Intercept)	2.00	1.41
Words (Intercept)	0.10	0.33

[1]Lg10WF=log10 word frequency

Note: Asterisks in the model formula denote interaction terms and main effects. Semicolons in the model output also show interactions (the common notation in the statistical package *R* was maintained).

Model 2

Formula

glmer(Accuracy~ Status+Noise*Predictability*Lg10WF+language_ability+WM+processing_speed + (1|Subject) + (1|Word), family='binomial', control=glmerControl(optimizer="bobyqa")))

Fixed Effects

**Predictor variable**	χ^2	**Df**	***p***
Group	12.5	1	.0004
Noise	702.5	1	< .0001
Predictability	1033.3	1	< .0001
language_ability	44.2	1	< .0001
Lg10WF	6.0	1	.0147
processing_speed	5.9	1	.0153
WM	0.0	1	.9773
Noise:Predictability	29.6	1	< .0001
Noise:language_ability	1.3	1	.2542
Predictability:language_ability	52.6	1	< .0001
Noise:Lg10WF	2.3	1	.1309
Predictability:Lg10WF	1.0	1	.3128
language_ability:Lg10WF	4.3	1	.0386
Noise:Predictability:language_ability	1.9	1	.1672
Noise:Predictability:Lg10WF	2.2	1	.1397
Noise:language_ability:Lg10WF	0.0	1	.8306
Predictability:language_ability:Lg10WF	0.2	1	.6658

Random Effects

**Variable**	**Variance**	**SD**
Subjects (Intercept)	2.00	1.42
Words (Intercept)	0.04	0.20

Model 3

Formula

glmer(Accuracy~ Noise*Predictability + Predictability*language_ability +language_ability *Lg10WF + Noise*language_ability + processing_speed + WMC + (1|Subject) + (1|Word), family='binomial', control=glmerControl(optimizer="bobyqa")))

Fixed Effects

**Predictor variable**	Bilinguals χ^2	**Df**	***p***	Monolinguals χ^2	**Df**	***p***
Noise	369.3	1	< .0001	343.5	1	< .0001
Predictability	449.7	1	< .0001	622.8	1	< .0001
language_ability	29.9	1	< .0001	12.9	1	0.0003
Lg10WF	8.6	1	.0034	3.3	1	0.0706
processing_speed	3.1	1	.0769	2.3	1	0.1290
WM	0.0	1	.8585	0.1	1	0.8174
Noise:Predictability	22.8	1	< .0001	11.6	1	0.0007
Noise: language_ability	0.0	1	.8855	0.0	1	0.8384
Predictability: language_ability	7.3	1	.0067	8.6	1	0.0033
language_ability :Lg10WF	0.8	1	.3680	0.1	1	0.7523

Random Effects

**Variable**	**Variance**	**SD**	**Variance**	**SD**
Subjects (Intercept)	1.83	1.35	2.25	1.50
Words (Intercept)	0.04	0.20	0.04	0.21

Correlation Matrix between different predictor and outcome variables

	Language ability	Age	Hearing (self-rated)	Involuntary Attention (from attention test)	High Noise Low Predictability	High Noise High Predictability	Low Noise High Predictability	Low Noise Low Predictability	Working Memory	Conflict resolution (from attention test)	Processing speed (from attention test)
Language ability		.13	.20	.45	< .01	< .00	< .00	< .00	< .00	.91	.73
Age	.15		.88	.29	.39	.17	.24	.53	.42	.20	.05
Hearing (self-rated)	.13	.01		.30	.79	.93	.32	.07	.08	.40	.49
Involuntary Attention (from attention test)	.08	-.11	.10		.80	.51	.64	.14	.89	.07	.79
High Noise Low Predictability	**.54**	.09	.03	.03		< .00	< .00	< .00	< .00	.26	.06
High Noise High Predictability	**.76**	.14	.01	-.07	**.54**		< .00	< .00	< .00	.48	.02
Low Noise High Predictability	**.69**	.12	.10	.05	**.49**	**.59**		< .00	< .00	.83	.08
Low Noise Low Predictability	**.52**	.06	.18	.15	**.42**	**.51**	**.54**		.05	.42	.28
Working Memory	**.53**	.08	.18	-.01	**.34**	**.45**	**.31**	**.20**		.98	.02
Conflict resolution (from attention test)	.01	-.13	-.08	.18	.11	.07	-.02	.08	.00		.06
Processing speed (from attention test)	-.03	**.20**	-.07	-.03	-.19	**-.23**	-.17	-.11	**-.23**	-.19	

Note. Upper half of the matrix shows *p*-values and lower half shows correlations. Bolded correlations are significant at the .05 level. The sample size was n=101 for all correlations.
